# Right coronary ostial atresia as a cause of arrhythmia and cardiogenic shock in a young woman: a case report

**DOI:** 10.1093/bjrcr/uaae049

**Published:** 2025-01-10

**Authors:** Velio Ascenti, Silvia Tresoldi, Caterina B Monti, Stefano Lucreziotti, Simone Soldi, Maurizio Cariati, Gianpaolo Carrafiello

**Affiliations:** Postgraduation School in Radiodiagnostics, Università degli Studi di Milano, Milan 20122, Italy; Department of Diagnostic Services, Diagnostic and Interventional Radiology Unit, ASST Santi Paolo e Carlo, Presidio San Paolo, Milan 20142, Italy; Postgraduation School in Radiodiagnostics, Università degli Studi di Milano, Milan 20122, Italy; Cardio Thoracic Vascular Department, Cardiology Unit, ASST Santi Paolo e Carlo, Presidio San Carlo, Milan 20153, Italy; Department of Diagnostic Services, Diagnostic and Interventional Radiology Unit, ASST Santi Paolo e Carlo, Presidio San Paolo, Milan 20142, Italy; Department of Diagnostic Services, Diagnostic and Interventional Radiology Unit, ASST Santi Paolo e Carlo, Presidio San Paolo, Milan 20142, Italy; Unità Operativa di Radiologia, Cà Granda Ospedale Maggiore Policlinico, Fondazione I.R.C.C.S., Milan 20122, Italy; Dipartimento di Scienze della Salute, Università degli Studi di Milano, Milan 20122, Italy

**Keywords:** Cardiac computed Tomography Coronary Angiography, Congenital Coronary Anomaly, Coronary Ostial Atresia

## Abstract

A 19-year-old woman presented to the emergency department with arrhythmia and signs of cardiogenic shock. After a 12-lead electrocardiogram ruled out acute myocardial infarction, and cardiac magnetic resonance showed no sign of cardiomyopathy, cardiac computed tomography angiography (CCTA) was performed, displaying ostial atresia of the right coronary artery. She was thus referred to a specialist centre for congenital cardiovascular disease, where an electrophysiological study observed an arrhythmogenic focus on the posteromedial papillary muscle, which was ablated, and she has been asymptomatic since. When dealing with patients presenting with arrhythmias or cardiogenic shock, and no signs of myocardial infarction or cardiomyopathy, performing CCTA to study the anatomy of the coronary arteries is vital.

## Clinical presentation

**Figure 1.**
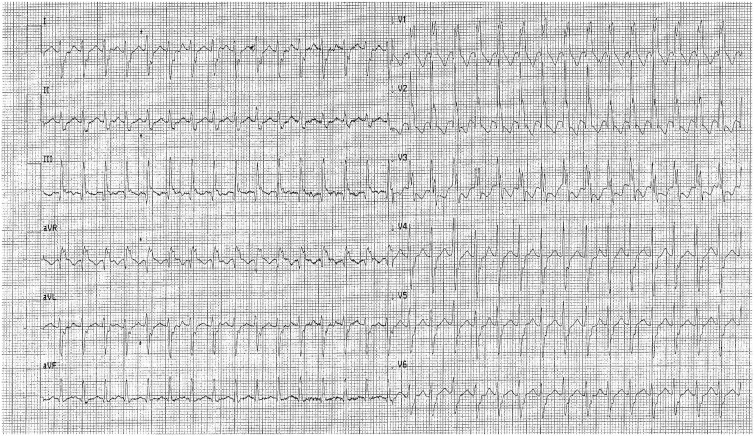
Twelve-lead electrocardiogram during palpitation showing ventricular tachycardia with right bundle branch block morphology and right axis (dominant S wave in leads I and aVL).

A 19-year-old woman presented to the emergency department of our institution for acute onset of palpitations. An electrocardiogram (ECG, Figure 1) showed ventricular tachycardia, with right branch bundle block associated to left posterior hemiblock, and T waves inversion in the inferior and precordial leads. Blood test showed elevated troponin (27 ng/L, normal values <14 ng/L) and NT-pro BNP (aminoterminal pro B-Type Natriuretic Peptide) levels (2225 pg/mL, normal values <130 pg/mL). Hence, she was admitted to the coronary care unit.

About 5 years before, she had already presented to the emergency department with cardiogenic shock due to fascicular ventricular tachycardia; she was subsequently hospitalized and underwent cardiac magnetic resonance (CMR) and a transoesophageal electrophysiological study, both with inconclusive results. She was discharged with a diagnosis of tachycardiomiopathy, with the prescription of a standard medical therapy (angiotensin-converting enzyme inhibitors, mineralocorticoid receptor antagonist, and beta blockers), and a follow-up was planned. Her subsequent clinical history was uneventful.

**Figure 2.**
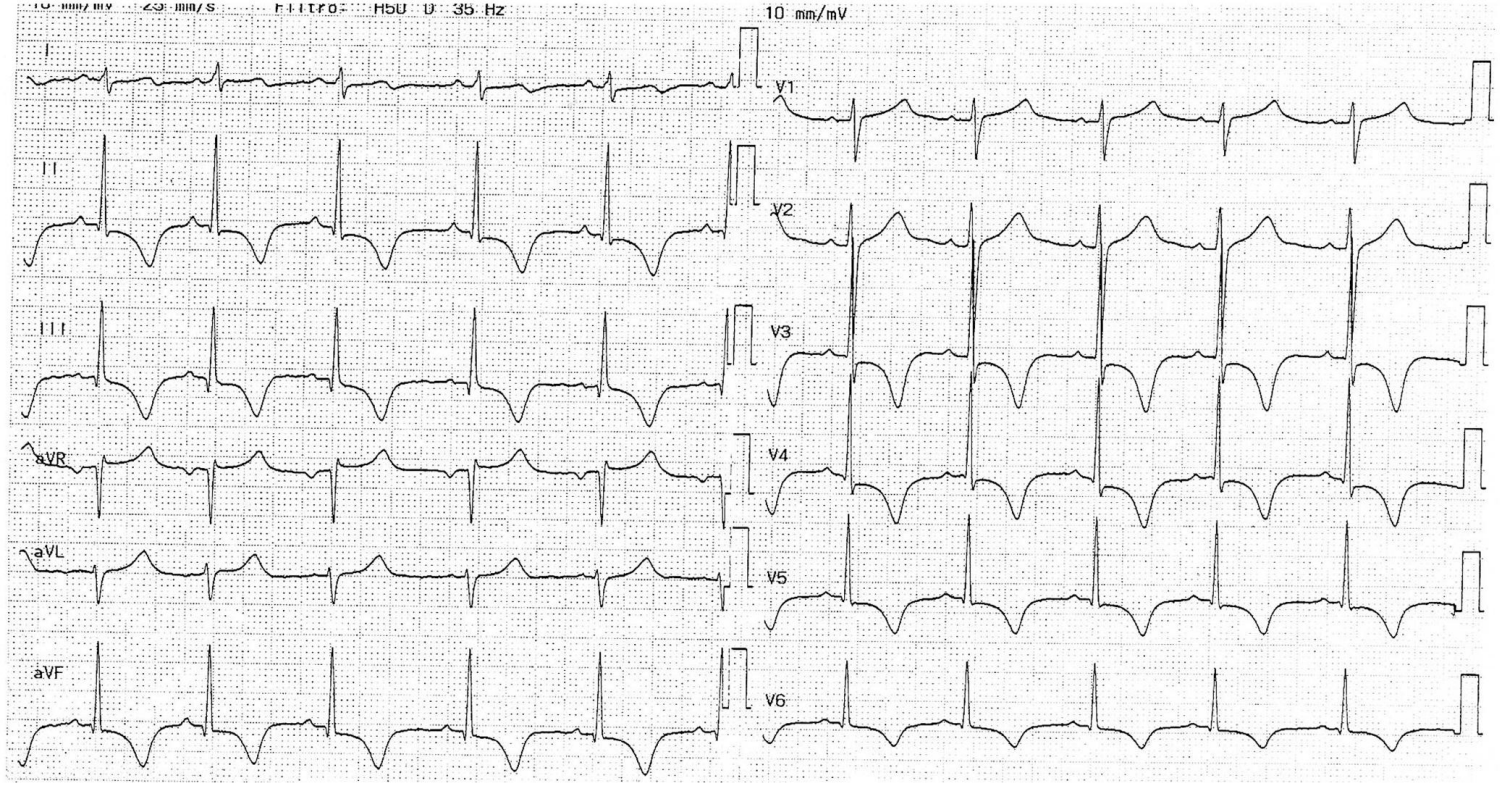
Basal 12-lead electrocardiogram showing sinus rhythm and abnormal ventricular repolarization with prolonged QT-interval and inversion of T waves in leads D2, D3, aVF, and V3-V6.

During the present hospitalization, no further episodes of hyperkinetic arrhythmias were detected. Basal 12-lead ECG is shown in Figure 2. Echocardiography showed diffuse hypokinesia of both the left and the right ventricles, and CMR was once again inconclusive. Then, the patient was advised to undergo cardiac computed tomography angiography (CCTA) to evaluate the anatomy of the coronary tree, under the suspicion of an undiagnosed congenital cardiac anomaly. CCTA was performed with a GE Lightspeed unit (GE HealthCare, Chicago, United States), with retrospective gating, at 100 kVp and 696 mAs, with a gantry rotation time of 0.35 s, and 0.625 mm slice thickness, with intravenous injection of 70 mL of Iomeron 400 mgI/mL (Bracco Imaging, Milan, Italy).

## Differential diagnosis

Cardiogenic shock, namely a shock caused by inadequate blood flow, may be caused by different pathologies such as myocardial infarction, arrhythmias, or other cardiomyopathies. Undiagnosed congenital heart disease is a non-negligible cause of cardiogenic shock in otherwise healthy adult patients. Once myocardial infarction is ruled out by a 12-lead ECG, and an underlying cardiomyopathy has been excluded by an inconclusive CMR examination, CCTA is the technique of choice for the differential diagnosis among diverse causes of cardiogenic shock.

## Investigations/imaging findings

**Figure 3.**
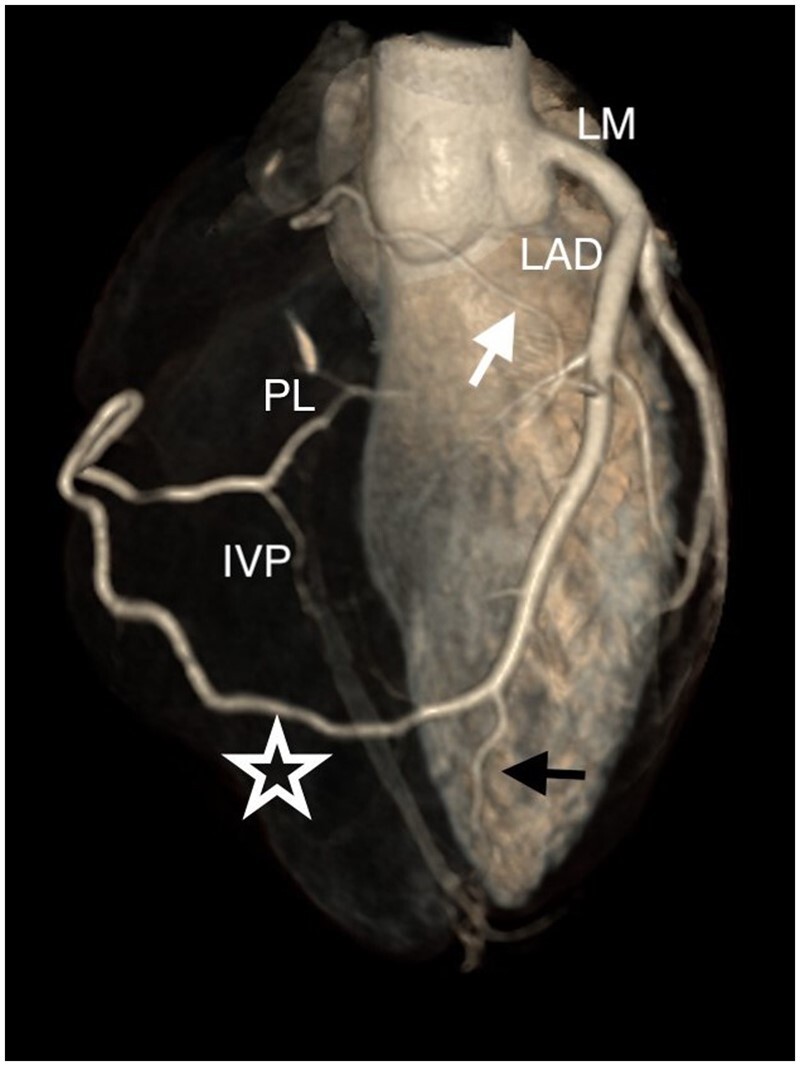
Three-dimensional volume rendering image, antero-superior view, showing the patient’s coronary tree, with a regular origin of the left main coronary artery from the aortic root, a left anterior descending artery giving origin first to the thin branch for the sinoatrial node (white arrow) and then to the wide branch (star) towards the right atrioventricular sulcus. The distal left anterior descending artery (black arrow) is also visible. No right coronary artery ostium is recognizable on the right coronary sinus. Abbreviations: IVP = posterior interventricular branch; PL = posterolateral branch.

**Figure 4.**
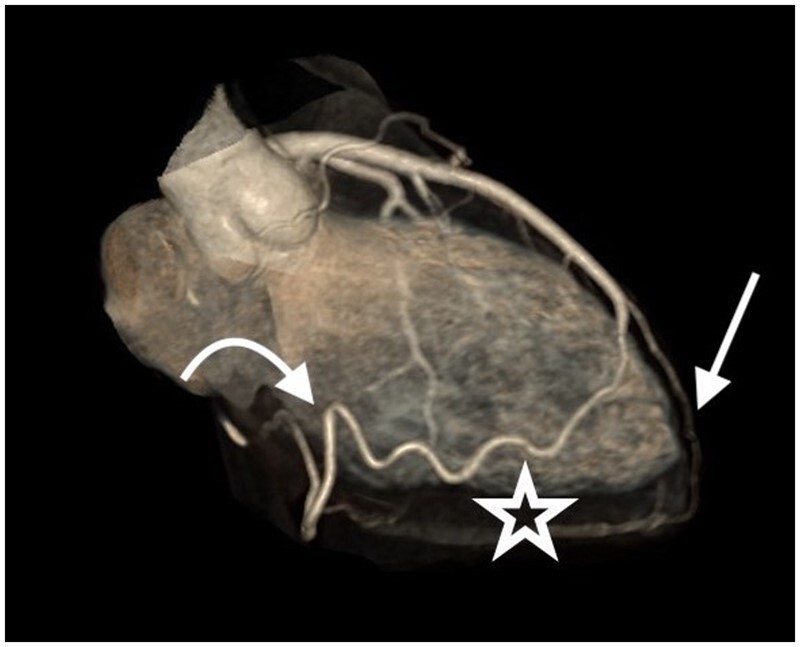
Three-dimensional volume rendering image, lateral view, showing the wide collateral branch (star) originating from the left anterior descending artery and reaching the right atrioventricular sulcus where it gives rise to the distal right coronary artery (curved arrow). The thin, distal left anterior descending artery (arrow) is also visible, reaching the cardiac apex.

**Figure 5.**
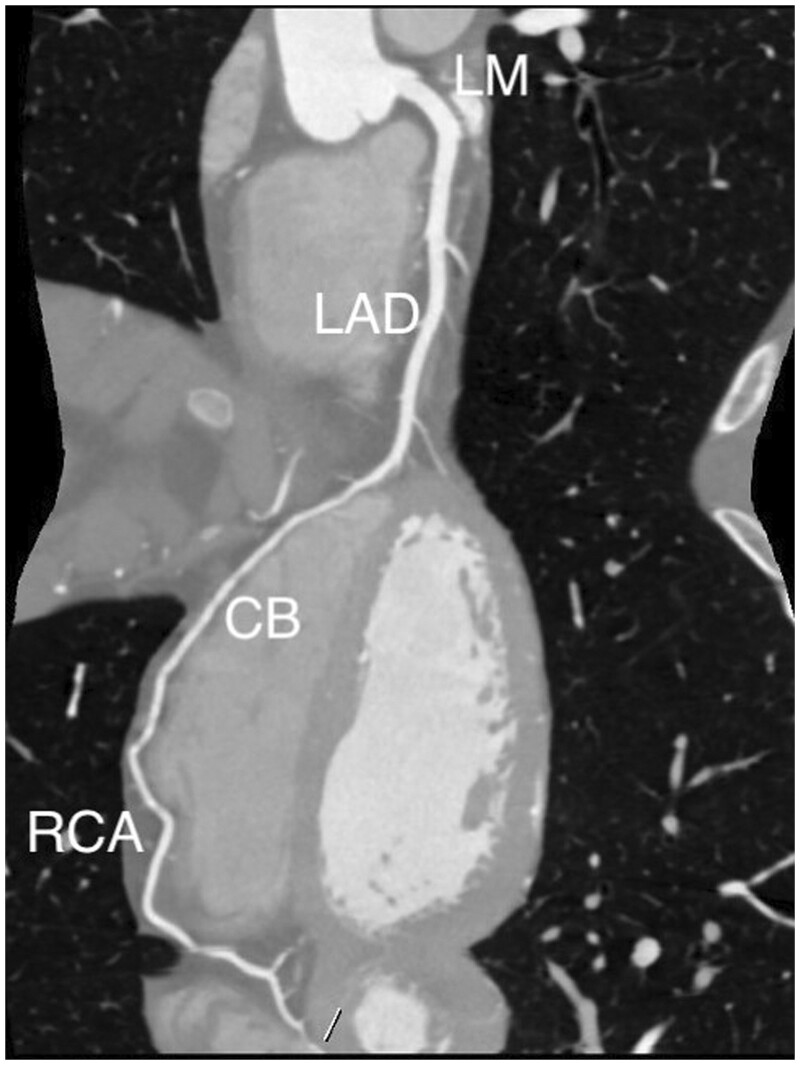
Curved multiplanar reformatted image displaying the entire course of the main coronary artery of the patient. Abbreviations: CB = collateral branch; LAD = left anterior descending artery; LM = left main coronary artery; RCA = right coronary artery.

The CCTA examination (Figures 3-5) showed regular origin of the left main coronary artery, the left anterior descending artery, which presented a wide ramus intermedium, 2 diagonal branches, and the left circumflex artery, which appeared thin and non-dominant. Among all these vessels, there was no evidence of obstructive coronary artery disease. However, the ostium and the proximal portion of the right coronary artery (RCA) were absent, whereas its mid and distal portions were supplied by a wide collateral branch originating from the distal left anterior descending artery, which then surrounded the lateral wall of the right ventricle, ran through the distal part of the atrioventricular groove, finally giving rise to thin posterior interventricular and posterolateral arteries. Furthermore, a thin branch going from the mid left anterior descending artery to the sinoatrial node was observed.

## Treatment, outcome, and follow-up

In view of the CCTA findings, the patient underwent an echo-stress test, which confirmed the presence of inducible myocardial ischaemia in the inferior wall, in line with the positioning of the arrhythmic focus. After being transferred to a specialized centre for the diagnosis and treatment of congenital heart disease, the patient underwent transcatheter radiofrequency ablation of sustained ventricular tachycardia originating by the posteromedial papillary muscle. Then, she was discharged with medical therapy (Acetylsalicylic acid and Verapamil). At her 6-month follow-up, she has always been asymptomatic, with no further signs of arrhythmias.

## Discussion

The RCA is one of the 2 main coronary vessels, which usually originates from the right aortic sinus and runs in the right portion of the atrioventricular groove, providing blood supply to the right atrium and ventricle, the interatrial and interventricular septa, the posteromedial papillary muscle, and vital areas such as the atrioventricular and sinoatrial nodes.

Congenital ostial atresia of a coronary artery is a condition where a single coronary artery, arising from the aortic trunk by a single coronary ostium, supplies the entire heart. This anomaly is assumed to be congenital, although progression in prenatal and postnatal life can occur, as there are only 1 or 2 connecting collaterals, without evidence of any narrowing at the transition between the supplying and the receiving vessels, in contrast to the rich network of collateral vessels seen in cases of acquired coronary artery occlusion. This rare congenital anomaly occurs as an isolated finding in approximately 0.024% of the population.^1^

As per the 1979 Lipton angiographic classification,^1^ a single coronary artery can be divided into 3 types according to the site of origin and anatomical distribution. The single coronary artery is called type R or L depending upon whether the ostium is located: if the right ostium is absent and the vessel originates from the left ostium, an L type is present. Conversely, an artery originating from the right ostium with no left ostium represents an R type. Subsequently, the single coronary artery is classified according to its anatomical distribution: group I indicates that a single coronary artery extends to supply the heart that should have been supplied by the course of the contralateral coronary artery, group II that one coronary artery rises from the proximal portion of another coronary artery, whereas group III indicates that the left anterior descending and circumflex coronary arteries arise separately.

Although the presence of a single coronary artery may potentially cause severe complications such as angina pectoris, cardiac arrhythmias, syncope, myocardial infarction, cardiogenic shock, or even sudden death, the presence of a collateral blood supply, especially at a young age with a low prevalence of cardiovascular risk factors, makes this condition often asymptomatic. However, coronary arteries anomalies may be isolated, but may also be associated with other cardiac defects or arrhythmias. Hence, overall clinical presentation depends on the severity of the impairment of myocardial perfusion due to anatomical anomalies.^2^

Ostial atresia of the RCA is an extremely rare variant of the single coronary artery, and it is usually asymptomatic, as patients with such anomaly tend to present with a well-developed collateral circulation rising from the larger left coronary artery. Hence, ostial atresia of the RCA most often represents an incidental finding in patients undergoing coronary arteriography or during autopsies.^3^ However, as the collateral blood supply may sometimes be insufficient, some individuals may develop serious clinical complications, related to the undersupplied territories pertaining to the RCA.^4–6^

In particular, the young woman in our case presented with ventricular tachycardia originating from the posteromedial papillary muscle, which belongs to the vascular territory of the RCA, likely due to an arrhythmic focus originating from an insufficient blood supply. Our patient presented with a suboptimal RCA vascularization, as she displayed a Lipton type L-I single coronary anomaly. Indeed, a study by Choudhury et al^7^ reported that the lack of an adequate blood supply from coronary anomalies may lead to ischaemia in the nodes and papillary muscle area, which can be followed by focal ischaemia fibrosis, leading to dysfunction and subsequent ECG alterations. A further explanation for her arrhythmia may be that ischaemia, due to RCA agenesis, can promote abnormal automaticity of Purkinje fibres, which are also found in the papillary muscles, that might cause ventricular fascicular tachycardia.^8^

In conclusion, coronary anomalies must always be considered when encountering young patients with arrhythmias or cardiogenic shock and no known evidence of congenital heart disease. In such cases, performing CCTA is vital as it represents the best non-invasive imaging technique in terms of sensibility, specificity, positive predictive value, and negative predictive value with regard to coronary anatomy.^9^^,^^10^

## Learning points

- Albeit rare and often asymptomatic, coronary artery anomalies may sometimes lead to important cardiovascular signs and symptoms, up to arrhythmias and cardiogenic shock.
- Even when no clear sign of myocardial infarction is present, an abnormal coronary anatomy may present with an underlying myocardial rearrangement due to a suboptimal blood supply, which may in turn generate arrhythmias.
- Once other causes of cardiovascular symptoms such as myocardial infarction or cardiomyopathies have been ruled out, cardiac computed tomography angiography is warranted to study the anatomy of the coronary circulation.
